# Radiomics analysis and classification of esophageal involvement in systemic sclerosis using chest CT images

**DOI:** 10.1007/s11548-026-03771-4

**Published:** 2026-08-03

**Authors:** Yuito Ueda, Shinnosuke Watanabe, Rea Ikeda, Mikito Suzuki, Masataka Kuwana, Hiroshi Takemura

**Affiliations:** 1 Department of Mechanical and Aerospace Engineering, Tokyo University of Science, 2641 Yamazaki, Noda-shi, Chiba 278-8510 Japan; 2 School of Medicine, Nippon Medical School, 1-1-5 Sendagi, Bunkyo-ku, Tokyo, 113-8602 Japan; 3 Department of Allergy and Rheumatology, Nippon Medical School Graduate School of Medicine, 1-1-5 Sendagi, Bunkyo-ku, Tokyo, 113-8602 Japan

**Keywords:** Systemic sclerosis (SSc), CT imaging, Radiomics, XGBoost

## Abstract

****Purpose**:**

Esophageal involvement is a common and early manifestation of systemic sclerosis (SSc). This involvement is mainly evaluated by esophageal manometry, but this test is invasive and provides limited morphological information. This study aimed to identify CT-based radiomics features related to esophageal involvement and to evaluate whether the presence or absence of esophageal involvement can be classified with high accuracy.

****Methods**:**

Chest CT images from 64 subjects were analyzed, including 30 patients with SSc and 34 normal subjects. The esophageal region was automatically segmented, and radiomics features were extracted from ellipse-fitted regions of interest on axial slices. Features showing significant differences were selected, and feature combinations were evaluated using exhaustive search. Classification was performed using XGBoost.

****Results**:**

First-order statistical features at levels corresponding to the third to fifth thoracic vertebrae were significantly associated with the presence of esophageal involvement. Classification using these features showed high accuracy when five to six features were used, indicating that the presence or absence of esophageal involvement can be identified accurately even with a small number of features.

****Conclusion**:**

CT-based radiomics analysis may be a useful noninvasive method for evaluating esophageal involvement. Future studies incorporating pathological correlation and surrounding tissue information may further improve clinical applicability.

## Introduction

Systemic sclerosis (SSc) is a rare autoimmune disease with many different features. It is characterized by skin hardening, vascular damage, and organ involvement, including the lungs [1, 2]. Although the prevalence of SSc is relatively low, the disease burden due to complications is high. It has been reported that SSc has the highest mortality rate among all rheumatic diseases [3]. There is no single established test to diagnose SSc. Therefore, the diagnosis is made by evaluating multiple findings together, including clinical features such as Raynaud’s phenomenon and sclerodactyly, according to classification criteria [4, 5].

Gastrointestinal involvement is one of the most common internal organ complications in SSc and is reported in more than 90% of patients [6]. Among these, esophageal involvement is very common and can appear at an early stage of the disease [7]. Esophageal manometry is the standard method to evaluate esophageal involvement, as it can quantitatively assess the resulting motility disorders. However, this test is invasive, does not provide morphological information, and has low patient acceptance [8].

In recent years, it has been reported that esophageal dysmotility is often found in SSc patients who show esophageal dilatation on high-resolution CT (HRCT). They suggested that morphological findings obtained from CT may help in the evaluation of esophageal function [8]. However, simple diameter measurement alone cannot fully capture the relationship with esophageal involvement. Therefore, more comprehensive and quantitative analysis methods using CT images are needed.

Recently, radiomics analysis, which extracts quantitative features such as shape, density distribution, and texture from CT images, has attracted attention. It is expected to be useful for diagnosis and disease assessment in many diseases [9–11].

Therefore, the aim of this study was to identify CT radiomics features related to esophageal involvement in SSc. We also examined whether the presence or absence of esophageal involvement can be classified with high accuracy using a small number of features for clinical application.

## Materials and measurements

### Study patients and CT image acquisition

Thirty patients diagnosed with SSc with esophageal involvement were defined as the abnormal group, and 34 patients without esophageal involvement were defined as the normal group. A total of 64 chest CT images were used. The normal group consisted of 15 patients diagnosed with SSc without esophageal involvement and 19 healthy subjects without SSc or esophageal involvement. The mean age was 59.8 ± 13.4 years (mean ± standard deviation) in the abnormal group and 58.9 ± 16.7 years in the normal group. Disease duration in SSc cases was expressed as the median. It was 27 months (range: 8.2–91 months) in the abnormal group and 12 months (range: 6.0–33 months) in SSc cases included in the normal group. Regarding subtypes, diffuse cutaneous SSc (dcSSc) included 16 cases and limited cutaneous SSc (lcSSc) included 14 cases. In the normal group, there were 8 dcSSc cases and 7 lcSSc cases.

The presence or absence of esophageal involvement was evaluated based on high-resolution esophageal manometry (HRM) performed at the Department of Gastroenterology and Hepatology, Nippon Medical School. The devices used were Starlet (Star Medical, Inc., Tokyo, Japan) and the Zvu HRiM system (Diversatek Inc., Milwaukee, USA). The evaluation indices were Integrated Relaxation Pressure (IRP), Distal Contractile Integral (DCI), and Distal Latency (DL). The results were classified into normal and abnormal groups based on these indices according to the Chicago Classification version 4.0. The abnormal group included ineffective esophageal motility (IEM) and absent contractility (AC) [12–14].

CT images were acquired with a matrix size of 512 × 512 pixels. The in-plane spatial resolution was 0.59–0.80 mm, and the slice thickness was 5.0 mm.

### Esophageal segmentation and ROI definition

**Fig. 1:**
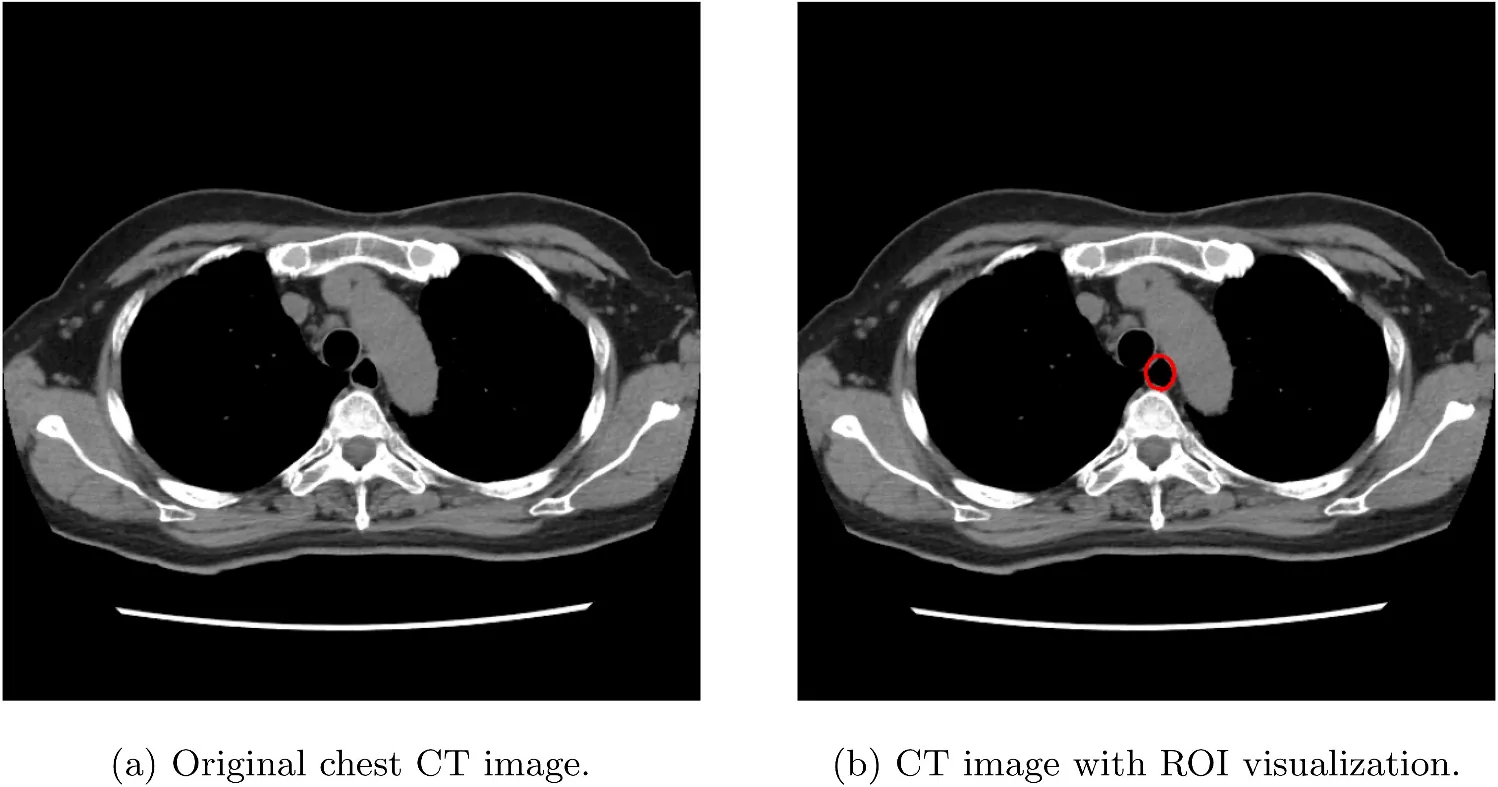
CT images used in this experiment

Automatic segmentation of the esophagus was performed on the chest CT images using TotalSegmentator [15]. For the segmented esophageal region, ellipse fitting was applied on each slice to define the region of interest (ROI). This approach was used to reduce the effect of shape errors caused by automatic segmentation and to allow stable evaluation of a region including the esophageal wall. Also, this method was based on anatomical knowledge that the esophageal cross section on axial images is often not circular but flattened in the anterior–posterior direction [16]. Figure 1 shows a CT image of a subject diagnosed with SSc and an image with ellipse fitting applied to the esophagus.

The esophagus is known to run through the thoracic cavity from the first thoracic vertebra (T1) to approximately the tenth to eleventh thoracic vertebrae (T10–T11) [16]. Therefore, in this study, CT images corresponding to the region from T1 to T12 were analyzed to comprehensively evaluate the entire thoracic esophagus. Slice selection was performed by one physician, and the analysis was conducted using the ROI mask images corresponding to the selected slices.

### Image preprocessing

Before radiomics analysis, the following preprocessing steps were performed. Pixel values of the CT images were expressed in Hounsfield units (HU), and no additional intensity normalization was applied. Considering that the slice thickness was 5 mm, the analysis was performed as a two-dimensional analysis for each axial slice. Although there were differences in in-plane spatial resolution among subjects, spatial resampling was not performed in order to preserve the original image information.

### Radiomics feature extraction

Radiomics features were extracted from the preprocessed CT images and the corresponding esophageal mask images using the PyRadiomics library. The bin width for gray-level quantization was set to 25 HU. In this study, only features calculated from the original images. A total of 102 features were extracted. These features consisted of shape features, first-order statistical features, and texture features.

## Statistical analysis

From each subject, 102 radiomics features were extracted from 12 slices corresponding to T1–T12, resulting in a total of 1,224 features. For the analysis, features that contained missing values were excluded.

For comparison between the SSc group with esophageal involvement and the normal group, the Mann–Whitney U test was performed for each feature. The significance level was set at 1% (p < 0.01). As a result, 208 features showed a significant difference between the two groups. In particular, many significant features were observed at the levels corresponding to T3–T5. At the T3, T4, and T5 levels, 42, 49, and 40 features were significant, respectively.

Among the 208 features with significant differences, the top 20 features with the smallest p values were selected as highly statistically significant. The selected features are shown in Table 1. Most of the selected features were classified as first-order statistical features, and some texture features were also included.

**Table 1:** Top 20 radiomic features showing significant differences between groups

Feature name	Thoracic level	Category	*p*-value
Median	T5	First-order	$4.19 \times 10^{-8}$
Mean	T4	First-order	$1.23 \times 10^{-6}$
Root mean squared	T4	First-order	$2.41 \times 10^{-6}$
Energy	T4	First-order	$2.94 \times 10^{-6}$
Mean	T5	First-order	$4.07 \times 10^{-6}$
Median	T4	First-order	$4.48 \times 10^{-6}$
Total energy	T4	First-order	$5.26 \times 10^{-6}$
10 Percentile	T4	First-order	$5.53 \times 10^{-6}$
10 Percentile	T5	First-order	$9.89 \times 10^{-6}$
Run entropy	T4	Texture	$1.12 \times 10^{-5}$
Zone entropy	T4	Texture	$1.12 \times 10^{-5}$
Median	T3	First-order	$1.52 \times 10^{-5}$
Root mean squared	T3	First-order	$1.72 \times 10^{-5}$
Inverse variance	T4	Texture	$2.19 \times 10^{-5}$
Energy	T3	First-order	$2.62 \times 10^{-5}$
10 Percentile	T3	First-order	$3.49 \times 10^{-5}$
Energy	T5	First-order	$3.96 \times 10^{-5}$
Run entropy	T3	Texture	$4.19 \times 10^{-5}$
Zone entropy	T3	Texture	$4.19 \times 10^{-5}$
Total energy	T3	First-order	$4.44 \times 10^{-5}$

## Construction and evaluation of classification models

### Exhaustive search of feature combinations

To further select features that are useful for discriminating the presence or absence of esophageal involvement, an analysis based on exhaustive search was performed. The exhaustive search method evaluates all possible combinations of candidate features and searches for models with high classification performance [17].

The objective variable was the presence of esophageal involvement (involvement present: 1, normal: 0). As explanatory variables, the top 20 radiomics features that showed significant differences in the statistical analysis described in the previous section were used. When the number of features is large, the number of possible combinations becomes very large. In addition, for practical clinical application, models using a small number of features are preferred. Therefore, in this study, the search was limited to combinations of six or fewer features.

### Model construction using XGBoost

XGBoost (eXtreme Gradient Boosting) is a gradient boosting method that uses decision trees as base learners. It can handle nonlinear relationships between features even with a small number of features and a small sample size. Therefore, XGBoost was used as the classification model in this study.

The hyperparameters of the XGBoost classifier were fixed for all feature combinations. The number of trees was set to 100, the maximum tree depth was set to 3, and the learning rate was set to 0.1.

Tenfold cross-validation was used for model evaluation. Stratified sampling was applied to keep the class ratio in each fold. Training and evaluation were performed in each fold of the cross-validation. Accuracy was used as the evaluation metric, and the mean accuracy obtained from the tenfold cross-validation was calculated as the classification performance for each feature combination.

### Classification performance and feature appearance trends

Among the 1335 feature combinations, accuracy greater than 85% was achieved. The highest classification performance was obtained with specific combinations using five or six features, with accuracy exceeding 89%. In addition, combinations using two features, such as the median at T5 and the root mean squared at T4, or three features including these two plus the 10 percentile at T5, also showed high accuracy of about 88%. These results indicate that high classification performance can be achieved even with a small number of features. Furthermore, a combination using only two features, the median and the energy at T5, focused only on features from T5 and still achieved an accuracy of about 83%.

**Fig. 2:**
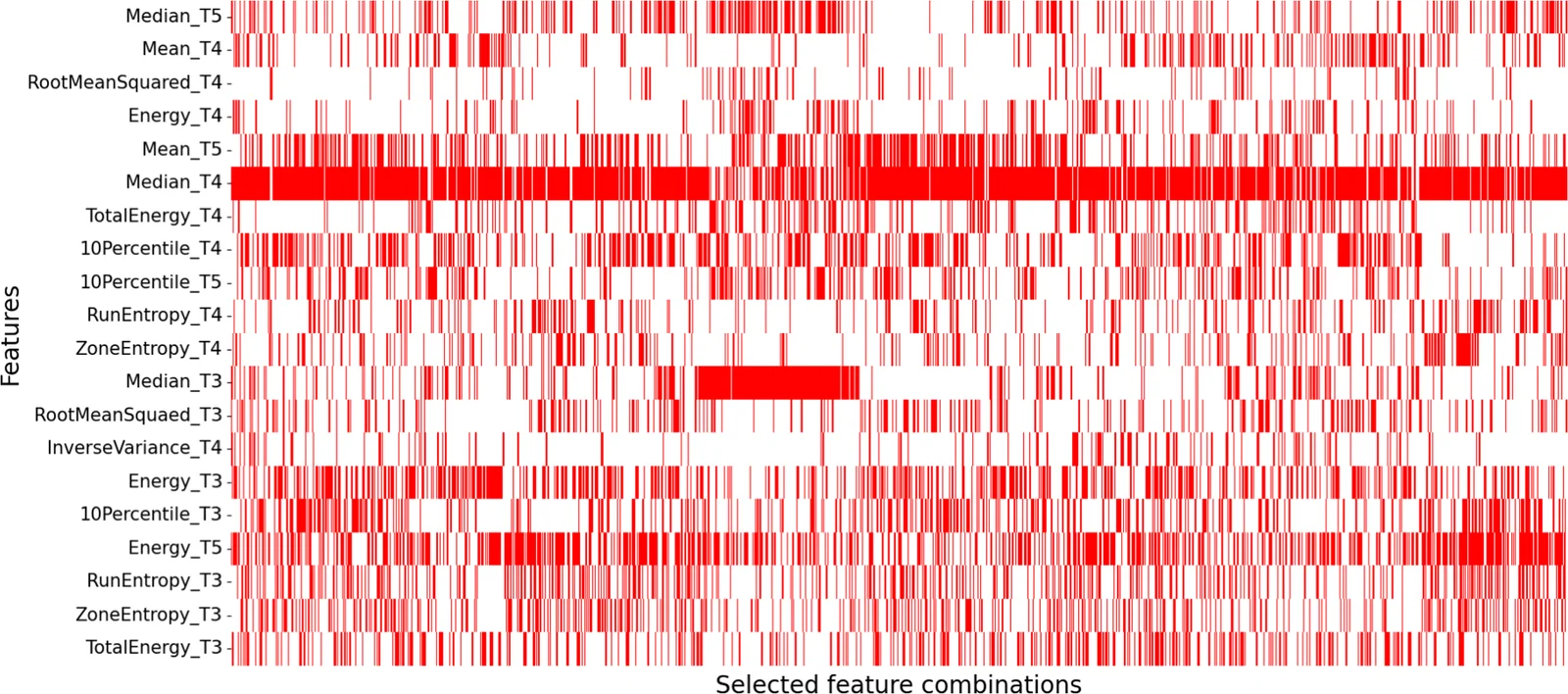
Appearance frequency of each feature

Next, we focused on the group of models with high classification performance and counted the appearance frequency of each feature. Figure 2 shows the distribution of feature appearance frequencies in the 1335 high-performance models with accuracy of 85% or higher. The median at T4 was the most frequently used feature and appeared in 1,168 combinations. This was followed by the energy at T5, which was used in 658 combinations. In contrast, the inverse variance and root mean squared at T4 were each used in fewer than 150 combinations.

## Discussion

### Discussion of the statistical analysis

From the results in “Statistical analysis” section, it was suggested that esophageal involvement associated with SSc tends to appear as image features in a relatively middle region of the thoracic esophagus. In particular, many features showing significant differences were observed in slices corresponding to T3–T5. This suggests that imaging changes related to this involvement may appear locally. These results indicate that analysis considering anatomical location is useful, rather than evaluating the entire esophagus in a uniform manner.

In addition, it is notable that many of the features showing significant differences were classified as first-order statistical features. First-order features reflect the distribution of pixel values within the ROI itself and do not evaluate spatial relationships between pixels, unlike texture features. This suggests that in esophageal involvement associated with SSc, changes in CT value distribution, such as shifts or variability in intensity levels, may be more apparent than heterogeneity of tissue structure.

In this study, the ROI was defined using ellipse fitting, and the analysis mainly focused on the region inside the esophageal wall. Therefore, surrounding tissues were not included in the evaluation, and it cannot be ruled out that the ROI setting influenced the results to some extent. In future studies, expanding the ROI to include surrounding tissues may allow the effects of esophageal involvement to be captured more comprehensively.

### Discussion of classification model construction and evaluation

The results in “Construction and evaluation of classification models” showed that the high classification performance can be achieved using XGBoost even under conditions with a small number of features. In particular, high accuracy was obtained in some cases when five or six features were used. This suggests that reliable discrimination is possible even with limited information. These findings indicate the potential for constructing models with a reduced number of features, which is important for clinical application.

In this study, candidate features were limited to those that showed significant differences in the statistical analysis, which partially constrained the selection trend. However, the fact that some features were consistently selected across multiple combinations suggests stability and consistency in feature selection for model construction. In particular, the median at T4 was frequently used in high-performance models, suggesting that it may be a feature that strongly contributes to discrimination under the conditions of this study.

### Limitations

In this study, we identified CT radiomics features associated with esophageal involvement in SSc and showed that first-order features, especially at the levels corresponding to the third to fifth thoracic vertebrae, were significantly related to its presence.

However, SSc is a rare disease, and the number of available cases is limited. In addition, only a limited number of institutions can perform specialized tests such as esophageal manometry. Therefore, there are constraints in case collection and clinical data acquisition, which limit the generalizability of the results. Furthermore, as this was a single-center study, there may be bias in-patient characteristics. Therefore, future studies with larger sample sizes and multicenter data are needed to validate these findings.

## Conclusion and future work

In this study, we identified CT image radiomics features associated with esophageal involvement in SSc and examined whether the presence or absence of this involvement can be classified under conditions using a small number of features. The statistical analysis showed that features mainly consisting of first-order statistics at the levels corresponding to T3–T5 were significantly associated with the presence of esophageal involvement. Furthermore, exhaustive search of feature combinations and classification using XGBoost demonstrated that some models using five to six features achieved high classification accuracy. These results suggest that the presence or absence of esophageal involvement can be classified with high accuracy even with a small number of features. In addition, the analysis of feature appearance frequency in high-performance models showed that first-order features at specific thoracic levels were repeatedly selected, suggesting that they reflect imaging characteristics of esophageal involvement associated with SSc.

In this study, the analysis was limited to extracting features that showed significant differences, and the factors explaining why these features are associated with the presence of esophageal involvement were not fully clarified. In future work, it will be necessary to improve the interpretability of these features by linking them with pathological findings and image physical properties. In addition, beyond ROIs limited to the esophageal wall, future analyses that include surrounding esophageal tissues may allow more comprehensive evaluation of imaging changes associated with esophageal involvement.

## References

[1] Bergamasco A, Hartmann N, Wallace L, Verpillat P (2019) Epidemiology of systemic sclerosis and systemic sclerosis-associated interstitial lung disease. Clin Epidemiol 11:257–273. 10.2147/CLEP.S191418 PMC6497473 31114386

[2] Smith V, Scirè CA, Talarico R, Airo P, Alexander T, Allanore Y, Bruni C, Codullo V, Dalm V, De Vries-Bouwstra J, Della Rossa A, Distler O, Galetti I, Launay D, Lepri G, Mathian A, Mouthon L, Ruaro B, Sulli A, Tincani A, Vandecasteele E, Vanhaecke A, Vanthuyne M, Hoogen F, Van Vollenhoven R, Voskuyl AE, Zanatta E, Bombardieri S, Burmester G, Fonseca JE, Frank C, Hachulla E, Houssiau F, Mueller-Ladner U, Schneider M, Laar JM, Vieira A, Cutolo M, Mosca M, Matucci-Cerinic M (2018) Systemic sclerosis: state of the art on clinical practice guidelines. RMD Open 4:000782. 10.1136/rmdopen-2018-000782 PMC6203100 30402270

[3] Volkmann ER, Andrèasson K, Smith V (2023) Systemic sclerosis. The Lancet 401(10373):304–318. 10.1016/S0140-6736(22)01692-0 PMC9892343 36442487

[4] Hachulla E, Launay D (2011) Diagnosis and classification of systemic sclerosis. Clin Rev Allergy Immunol 40:78–83. 10.1007/s12016-010-8198-y 20143182

[5] Hoogen F, Khanna D, Fransen J, Johnson SR, Baron M, Tyndall A, Matucci-Cerinic M, Naden RP, Medsger TA Jr, Carreira PE, Riemekasten G, Clements PJ, Denton CP, Distler O, Allanore Y, Furst DE, Gabrielli A, Mayes MD, Laar JM, Seibold JR, Czirjak L, Steen VD, Inanc M, Kowal-Bielecka O, Müller-Ladner U, Valentini G, Veale DJ, Vonk MC, Walker UA, Chung L, Collier DH, Csuka ME, Fessler BJ, Guiducci S, Herrick A, Hsu VM, Jimenez S, Kahaleh B, Merkel PA, Sierakowski S, Silver RM, Simms RW, Varga J, Pope JE (2013) 2013 classification criteria for systemic sclerosis: an american college of rheumatology/european league against rheumatism collaborative initiative. Ann Rheum Dis 72(11):1743–1755. 10.1136/annrheumdis-2013-204424 24092682

[6] McMahan ZH (2019) Gastrointestinal involvement in systemic sclerosis: an update. Rheumatology (Oxford) 31(6):561–568. 10.1097/BOR.0000000000000645 PMC10266535 31389815

[7] Zampatti N, Garaiman A, Jordan S, Dobrota R, Becker MO, Maurer B, Distler O, Mihai C (2021) Performance of the ucla scleroderma clinical trials consortium gastrointestinal tract 2.0 instrument as a clinical decision aid in the routine clinical care of patients with systemic sclerosis. Arthritis Res. Ther. 23(125) 10.1186/s13075-021-02506-x PMC8061014 33888149

[8] Pitrez EH, Bredemeier M, Xavier RM, Capobianco KG, Restelli VG, Vieira MV, Ludwig DHC, Brenol JCT, Furtado APA, Fonseca LMB, Gutfilen B (2006) Oesophageal dysmotility in systemic sclerosis: comparison of hrct and scintigraphy. Br J Radiol 79(945):719–724. 10.1259/bjr/17000205 16885178

[9] Gillies RJ, Kinahan PE, Hricak H (2016) Radiomics: Images are more than pictures, they are data. Radiology 278(2):563–577. 10.1148/radiol.2015151169 PMC4734157 26579733

[10] Martini K, Baessler B, Bogowicz M, Blüthgen C, Mannil M, Tanadini-Lang S, Schniering J, Maurer B, Frauenfelder T (2021) Applicability of radiomics in interstitial lung disease associated with systemic sclerosis: proof of concept. Eur Radiol 31:1987–1998 10.1007/s00330-020-07293-8 PMC7979612 33025174

[11] Schniering J, Maciukiewicz M, Gabrys HS, Brunner M, Blüthgen C, Meier C, Braga-Lagache S, Uldry A-C, Heller M, Guckenberger M, Fretheim H, Nakas CT, Hoffmann-Vold A-M, Distler O, Frauenfelder T, Tanadini-Lang S, Maurer B (2022) Computed tomography-based radiomics decodes prognostic and molecular differences in interstitial lung disease related to systemic sclerosis. Eur Respir J 59(5):2004503. 10.1183/13993003.04503-2020 PMC9117734 34649979

[12] Yadlapati R, Kahrilas PJ, Fox MR, Bredenoord AJ, Gyawali CP, Roman S, Babaei A, Mittal RK, Rommel N, Savarino E, Sifrim D, Smout A, Vaezi MF, Zerbib F, Akiyama J, Bhatia S, Bor S, Carlson DA, Chen JW, Cisternas D, Cock C, Coss-Adame E, Bortoli N, Defilippi C, Fass R, Ghoshal UC, Gonlachanvit S, Hani A, Hebbard GS, Jung KW, Katz P, Katzka DA, Khan A, Kohn GP, Lazarescu A, Lengliner J, Mittal SK, Omari T, Park MI, Penagini R, Pohl D, Richter JE, Serra J, Sweis R, Tack J, Tatum RP, Tutuian R, Vela MF, Wong RK, Wu JC, Xiao Y, Pandolfino JE (2021) Esophageal motility disorders on high-resolution manometry: Chicago classification version 4.0. Neurogastroenterol Motil 33(1), 14058 10.1111/nmo.14058 PMC8034247 33373111

[13] Kuribayashi S, Iwakiri K, Kawada A, Kawami N, Hoshino S, Takenouchi N, Hosaka H, Shimoyama Y, Kawamura O, Yamada M, Kusano M (2015) Variant parameter values-as defined by the chicago criteria-produced by manoscan and a new system with unisensor catheter. Neurogastroenterol Motil 27(2):188–194. 10.1111/nmo.12446 25271562

[14] Kuribayashi S, Iwakiri K, Shinozaki T, Hosaka H, Kawada A, Kawami N, Hoshino S, Takenouchi N, Shimoyama Y, Kawamura O, Kusano M, Uraoka T (2019) Clinical impact of different cut-off values in high-resolution manometry systems on diagnosing esophageal motility disorders. J Gastroenterol 54(12):1078–1082. 10.1007/s00535-019-01608-3 31388756

[15] Wasserthal J, Breit H-C, Meyer MT, Pradella M, Hinck D, Sauter AW, Heye T, Boll DT, Cyriac J, Yang S, Bach M, Segeroth M (2023) Totalsegmentator: Robust segmentation of 104 anatomic structures in ct images. Radiol Artif Intell 5(5) 10.1148/ryai.230024 PMC10546353 37795137

[16] Kuo B, Urma D (2006) Esophagus - anatomy and development. GI Motil Online. 10.1038/gimo6

[17] Igarashi Y, Ichikawa H, Nakanishi-Ohno Y, Takenaka H, Kawabata D, Eifuku S, Tamura R, Nagata K, Okada M (2018) Es-dos: Exhaustive search and density-of-states estimation as a general framework for sparse variable selection. J Phys: Conf Ser 1036:10–13. 10.1088/1742-6596/1036/1/012001

